# Antioxidant effect of a polyphenol-rich grape pomace extract on motility, viability and lipid peroxidation of thawed bovine spermatozoa

**DOI:** 10.1186/2241-5793-21-19

**Published:** 2014-11-03

**Authors:** Vasiliki G Sapanidou, Ioannis Margaritis, Nektarios Siahos, Konstantinos Arsenopoulos, Eleni Dragatidou, Ioannis A Taitzoglou, Ioannis A Zervos, Alexandros Theodoridis, Maria P Tsantarliotou

**Affiliations:** Laboratory of Physiology, School of Veterinary Medicine, Faculty of Health Science, Aristotle University of Thessaloniki, 54124 Thessaloniki, Greece; Laboratory of Animal Production Economics, School of Veterinary Medicine, Faculty of Health Science, Aristotle University of Thessaloniki, 54124 Thessaloniki, Greece

**Keywords:** Motility, Viability, Lipid peroxidation, Spermatozoa, Grape, Polyphenols

## Abstract

**Background:**

Grape extracts of the Greek species *Vitis vinifera* possess potent antioxidant properties *in vitro*. The freeze/thaw process and the preparation of semen during assisted reproductive techniques can adversely affect the functional integrity of spermatozoa. The objective was to assess the effect of three different concentrations (1 μg ml^−1^, 2 μg ml^−1^ and 5 μg ml^−1^) of a polyphenol-rich grape pomace extract on motility, viability, acrosomal and lipid peroxidation status of thawed bovine spermatozoa after 2 and 4 hrs of incubation.

**Results:**

The results indicate that the percentage of “Rapid” spermatozoa remained significantly increased (*p* <0.05) in the presence of 5 μg ml^−1^ of the extract, compared to the control after 2 hrs of incubation. Additionally, the incubation of spermatozoa with 2 μg ml^−1^ and 5 μg ml^−1^ of the extract for 2 hrs resulted in a significantly better maintenance of viable spermatozoa with intact acrosome (*p* <0.05). The other parameters did not show statistically significant changes. Moreover, the presence of 2 μg ml^−1^ and 5 μg ml^−1^ of the extract kept the levels of Malondialdehyde (MDA) production in significantly lower level, compared to the other groups, after 2 hrs and 4 hrs of incubation (*p* <0.05). Particularly, a dose-dependent effect was noticed after 2 hrs of incubation.

**Conclusions:**

Our results suggest that the grape pomace extract exerts a powerful antioxidant role, by suppressing lipid peroxidation, and provides protection in terms of motility and acrosomal integrity, which are correlated with *in vivo* fertility. The optimal extract concentration is 5 μg ml^−1^.

## Background

Motility is an essential property of fertile spermatozoa in order to traverse the female reproductive tract, reach the site of fertilization and penetrate the *zona pellucida* of the oocyte [1]. The most efficient method to estimate fertility is to accomplish *in vitro* fertilization, which is time consuming and expensive [2]. Besides, other groups suggest that simple laboratory techniques such as motility assessment, DNA fragmentation index and evaluation of plasma membrane integrity would be more advantageous [3]. Computer Assisted Sperm Analysis (CASA) is a non-subjective analyzer which provides the opportunity to assess multiple characteristics with repeatability. It is noteworthy that the total number of motile spermatozoa tended to be higher in high fertility bulls [4] while the evaluation of kinematic parameters obtained by CASA are highly correlated with higher *in vivo* fertility [5]. Moreover, successful fertilization predisposes that all the membranes of spermatozoon are intact [4], otherwise, fertilization would be compromised. For this reason, the membrane status is an indicator of sperm competency.

Motile and fertile spermatozoa during their aerobic metabolism produce physiological amounts of reactive oxygen species (ROS) [6]. Low levels of ROS are essential for hyperactivation motility, capacitation/acrosome reaction and other physiological processes [7]. However, spermatozoa are particularly susceptible to oxidative injury due to the abundance of plasma membrane polyunsaturated fatty acids [8]. These unsaturated fatty acids provide fluidity that is necessary for membrane fusion events (e.g., the acrosome reaction and sperm-egg interaction) and for sperm motility. However, the unsaturated nature of these molecules predisposes them to free radical attack and ongoing lipid peroxidation (LPO) throughout the sperm plasma membrane. This phenomenon has deleterious effects on the fluidity, integrity and flexibility of sperm plasma membrane, characteristics associated with fertilizing capacity. Once this process has been initiated, accumulation of lipid peroxides occurs on the sperm surface (resulting in loss of sperm motility) and oxidative damage to DNA can ensue [9]. LPO is known to be an informative and diagnostic tool to evaluate the membrane integrity [10].

Thawed mammalian spermatozoa do not contain sufficient mechanisms to counteract ROS because the seminal plasma, which is enriched with antioxidant compounds, is removed during the preparation of sperm for *in vitro* fertilization [11]. Cryopreservation is associated with ROS production, which leads to lipid peroxidation, resulting in loss of motility and membrane integrity [12]. On this basis, several *in vitro* studies have shown a positive effect of antioxidant supplementation on sperm characterisrics. Among the antioxidants employed *in vitro*, various plant-derived ones have been used after thawing, such as crocin from *Crocus sativus* L. [13, 14].

Grape extracts are considered as potent antioxidants and free radical scavengers, as shown in several studies [15]. The antioxidant properties of the polyphenolic compounds that grapes possess are responsible for the recently growing interest in the biological importance of grape extracts [16]. Polyphenols are secondary metabolites of plants, which are divided in two main categories, namely flavonoids and nonflavonoids. They exert a powerful role in absorption and neutralization of ROS [17]. Among the most common polyphenols found in grape extracts are *trans*-resveratrol, catechin, epicatechin, quercetin and gallic acid [18]. During the last decade, research has demonstrated that several grape extracts of the grape species *Vitis vinifera* possess potent antioxidant properties *in vitro,* as they scavenge several free radicals, such as 1,1-diphenyl-2-picrylhydrazyl (DPPH^•^), 2,2-Azino-bis-(3-ethyl-benzthiazoline-sulphonic acid (ABTS^•+^), superoxide (O_2_^•-^), hydroxyl (OH^•^), and peroxyl (ROO^•^) radicals [19]. Thus, the aim of the present study was to examine whether a polyphenol-rich grape pomace extract would have any effect on several motility parameters, estimated by CASA, on viability, acrosomal status and lipid peroxidaton level of thawed bovine spermatozoa in the media of Assisted Reproductive Techniques (ART) settings.

## Results

### Motility

The results are presented in Table 1, which includes semen features regarding motility. The percentage of “rapid” spermatozoa remained increased after incubation with 5 μg ml^−1^ of the grape extract compared to the control group at 2 hrs of incubation (*p* = 0.017). The “medium” and “slow” movement did not show any statistically significant differences (data not shown). Progressive motility and the velocity parameters VCL, VSL, VAP, as well as ALH (for abbreviations, see Methods) did not show any statistically significant differences in each time point (*p* >0.05).

**Table 1:** **The effect of different doses of the grape extract on CASA parameters (mean ± SD)**

Time	Conc. of grape extract	Total motile	Rapid	Progressive motile	VSL	VCL	VAP	ALH
(hrs)	(μg ml ^−1^)	(%)	(%)	(%)	(μm sec ^−1^)	(μm sec ^−1^)	(μm sec ^−1^)	(μm)
0	0	69.17 ± 5.16	50.50 ± 5.15	17.85 ± 4.53	21.25 ± 4.89	57.93 ± 2.97	31.28 ± 5.62	2.35 ± 0.30
	1	77.97 ± 5.38	51.70 ± 6.27	20.10 ± 5.74	20.53 ± 4.32	63.0 ± 8.28	36.13 ± 3.97	2.88 ± 0.39
	2	79.6 ± 5.36	56.20 ± 1.50	21.15 ± 4.84	23.73 ± 7.79	63.8 ± 3.84	37.53 ± 2.87	3.28 ± 0.57
	5	81.07 ± 3.7	56.25 ± 2.21	23.52 ± 1.64	25.15 ± 11.27	64.65 ± 2.3	43.88 ± 6.8	2.78 ± 0.15
2	0	45.65 ± 17.61	19.02 ± 6.22^a^	14.70 ± 3.98	29.8 ± 16.37	54.48 ± 14.06	38.25 ± 13.84	3.08 ± 0.31
	1	64.47 ± 16.03	31.57 ± 10.16^ab^	22.57 ± 8.14	24.0 ± 8.89	57.85 ± 3.88	39.0 ± 5.10	2.78 ± 0.15
	2	63.82 ± 9.53	38.57 ± 5.57^ab^	22.32 ± 7.48	33.15 ± 12.03	63.10 ± 14.66	42.25 ± 14.42	3.2 ± 0.16
	5	71.85 ± 3.61	44.15 ± 11.60^b^	26.07 ± 3.06	28.48 ± 16.06	63.83 ± 8.23	39.88 ± 5.67	3.12 ± 0.46
4	0	36.23 ± 6.97	13.00 ± 3.94	12.57 ± 2.00	19.48 ± 6.91	47.68 ± 8.14	28.38 ± 4.07	2.52 ± 0.58
	1	40.05 ± 13.04	16.47 ± 7.78	10.22 ± 4.77	24.45 ± 9.82	45.53 ± 9.73	25.88 ± 4.51	2.50 ± 0.58
	2	38.7 ± 4.86	15.78 ± 5.57	12.30 ± 3.56	19.48 ± 8.38	44.93 ± 9.35	25.18 ± 6.19	2.10 ± 0.84
	5	51.40 ± 6.22	25.52 ± 7.59	15.97 ± 3.25	19.78 ± 10.45	51.93 ± 10.54	28.70 ± 4.25	2.85 ± 0.70

^a,b^ Different letters in each treatment indicate statistically significant differences within each given time point (*p* <0.05, n = 4).

### Viability

Table 2 summarizes the results from the evaluation of the viability and the acrosomal status of spermatozoa, treated with different concentrations of grape extract (1 μg ml^−1^, 2 μg ml^−1^, 5 μg ml^−1^), in the presence of a negative control. Data showed that the incubation of spermatozoa with 2 μg ml^−1^ and 5 μg ml^−1^ of the grape extract for 2 hrs kept sperm viability higher compared to the other groups (*p* <0.05). Furthermore, the co-incubation of spermatozoa with 5 μg ml^−1^ of the grape extract maintained almost the same percentage of alive spermatozoa with intact acrosome after 4 hrs of incubation. These data indicate that there is no effect on the acrosomal integrity of the spermatozoa due to the presence of the grape extract. Finally, neither other grape extract concentration nor incubation time affected viability and acrosomal status of the samples evaluated.

**Table 2:** **The effect of different doses of the grape extract on sperm viability (mean ± SD)**

Time (hours)	Extract (μg ml ^−1^)	Alive spermatozoa-intact acrosome (%)
0	0	81.0 ± 2.4
	1	77.0 ± 3.0
	2	82.0 ± 1.8
	5	79.0 ± 3.2
2	0	49.0 ± 2.5^a^
	1	48.0 ± 1.8^a^
	2	54.5 ± 1.5^b^
	5	55.6 ± 1.5^b^
4	0	39.5 ± 2.9
	1	39.0 ± 5.4
	2	37.3 ± 2.6
	5	47.4 ± 13.5

Different letters in each column indicate statistically significant difference between the treatments within each given time point (*p* <0.05, n = 4).

### Measurement of lipid peroxidation

**Figure 1:**
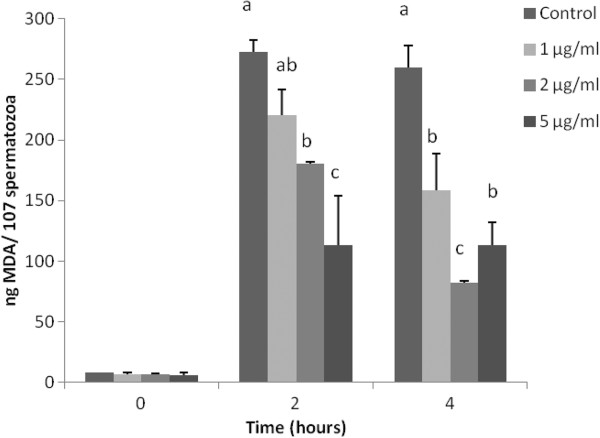
**The effect of the grape extract on LPO production of sperm (mean ± SD).** Different letters indicate statistically significant differences between different concentrations within each time point (*p* <0.05).

Figure 1 summarizes the results of the TBARS assay. Corresponding to supplementation, all the groups treated with the grape pomace extract had lower MDA production compared to the control, significantly or not. During 2 hrs of incubation, a dose-dependent reduction in MDA levels was observed. Moreover, statistically significant differences were noticed between: i) control and 2 μg ml^−1^ (*p* = 0.002), ii) control and 5 μg ml^−1^ (*p* <0.001), iii) 1 μg ml^−1^ and 5 μg ml^−1^ (*p* = 0.001) and iv) 5 μg ml^−1^ and all groups (*p* <0.03). After 4 hrs of incubation, the maximum antioxidant effect was observed in the presence of 2 μg ml^−1^ compared to all the other groups, while 5 μg ml^−1^ decreased the production of MDA significantly, compared to the control group (*p* <0.05). Nevertheless, no statistically significant difference was observed between 2 μg ml^−1^ and 5 μg ml^−1^ after 4 hrs of incubation (*p* >0.05).

## Discussion

In the present study, the co-incubation of thawed bovine sperm with 5 μg ml^−1^ of the polyphenol-rich grape pomace extract, maintained the percentage of rapid spermatozoa compared to the control after 2 hrs of sperm incubation. In our experiment we did not notice any influence of the grape extract on CASA kinematic parameters like VCL, VSL, VAP and ALH. The addition of 5 μg ml^−1^ of the polyphenol-rich grape pomace extract protected spermatozoa from lipid peroxidation after 2 hrs of incubation more effectively compared to all the other groups. LPO can cause protein oxidation which leads to loss of motility [20, 21]. The detection of high levels of MDA is negatively correlated with the motility parameters and the ability of the spermatozoa to penetrate the *zona pellucida* [22]. In conclusion, as for motility, the beneficial effect of the polyphenol-rich grape pomace extract at the concentration of 5 μg ml^−1^ may be due to the protective properties it exerts against LPO.

Regarding viability, the incubation of sperm supplemented with 2 μg ml^−1^ or 5 μg ml^−1^ of the extract, resulted in a significantly better maintenance of viable spermatozoa at 2 hrs of incubation, compared to all other groups, without showing any negative effect on acrosomal integrity. Several authors reported that fertility is positively correlated to plasma membrane integrity and progressive motility [3, 23].

However, there is evidence that the presence of some antioxidants (e.g. ascorbic acid and vitamine E) correlates beneficially with sperm characteristics such as motility, viability and lower production of MDA levels [24–26]. Polyphenols exert a significant antioxidant role due to their oxidoreduction properties. For this reason, they are capable of reducing lipid peroxidation in sperm [27]. The grape pomace extract used in the present study has a total polyphenol content equal to 648 mg of gallic acid per g of extract [28]. In our experiment, the incubation of spermatozoa with 2 μg ml^−1^ and 5 μg ml^−1^ resulted in an effective protection of the membrane’s phopsholipids after 2 hrs of incubation, while the former concentration suppressed the levels of MDA after 4 hrs of incubation. Furthermore, it has been reported that polyphenols increase cell viability, decrease intracellular Ca^2+^ levels and ROS formation and improve mitochondrial membrane potential in cells [29]. More specifically, quercetin and resveratrol affect intracellular calcium release, which can be observed through enhanced or sustained motility during storage or incubation without loss of sperm function. Nevertheless, the addition of resveratrol or quercetin before cryopreservation did not significantly affect progressive motility and plasma or membrane integrity of ovine spermatozoa [30]. In general, the action of phenolic compounds depends on their structure, dose, and method of administration as well as the half-life of the radical to be intercepted [31]. Our data indicate that the grape pomace extract used, inhibited the peroxidative damage in sperm and showed a dose-dependent reduction in MDA production after 2 hrs of incubation when different concentrations added to sperm samples. Finally, despite the fact that 2 μg ml^−1^ resulted in a significantly lower reduction of LPO after 4 hrs, spermatozoa that were treated with this concentration failed to maintain not only the total and progressive motility, but also the viability and acrosomal integrity. It is suggested that mild and low oxidative stress may enhance the fertilizing potential by promoting hyperactivation motility and capacitation, through increased tyrosine phosphorylation [32]. On the other hand, it should be noted that mild LPO predisposes the cell to capacitation/acrosome reaction and that extensive LPO is significantly correlated with impaired embryo development [33].

## Conclusion

In conclusion, the polyphenol-rich grape pomace extract used in our study provided protection to thawed spermatozoa against oxidative damage by suppressing lipid peroxidation and consequently maintained essential sperm characteristics, such as motility, viability and acrosomal integrity. In our experiments we showed that the optimal extract concentration that is capable of maintaining the functional integrity of spermatozoa is 5 μg ml^−1^. Further research should be held to investigate these effects on pre-freezing sperm as well as during the freezing-thawing process.

## Methods

Samples from frozen-thawed spermatozoa of four different mature bulls of proven fertility were used in this experiment. The samples were kindly offered by the Center of Artificial Insemination of Thessaloniki (Nea Ionia, Greece). The same four bulls were used in all replicates. Straws were thawed (37°C for 40 sec) and their contents were pooled in a conical tube. The samples were washed with 3× volume of Sperm Talp (100 mM NaCl, 3.1 mM KCl, 25 mM NaHCO_3_, 0.29 mM NaH_2_PO_4,_ 21.6 mM Na Lactate, 2 mM CaCl_2_, 1.5 mM MgCl_2_, 10 mM Hepes supplemented with 0.6% bovine serum, 1 mM sodium pyruvate and 50 μg ml^−1^ gentamycin) and centrifuged at 1000 rpm for 10 min. The procedure was repeated twice. Sperm concentration was determined with a haemocytometer (Optik Labor, Grale HDS, New South Wales, Australia).

The washed pool of spermatozoa was divided in four tubes and Sperm Talp was added in order to achieve a concentration of 20 × 10^6^ cells ml^−1^ in each tube. A tube was served as control, while the others were supplemented with four different concentrations of a polyphenol-rich grape pomace extract (1 μg ml^−1^, 2 μg ml^−1^, 5 μg ml^−1^). The concentrations were determined after a series of pre-experiments. The samples were analyzed in terms of motility, viability, acrosomal status and lipid peroxidation level immediately after sperm preparation (time point 0 hr) and at 2 hrs and 4 hrs incubation at 37°C. The experiment was repeated 4 times (n = 4).

The study was conducted under Good Laboratory Practice (GLP) conditions and the approval of the Research Ethical Committee of Faculty of Veterinary Medicine, 5/2013, Aristotle University.

### The grape extract

The grape pomace used belongs to the species *Vitis vinifera* and to the variety Batiki Tyrnavou (white grapes grown in Central Greece). The raw material was dried in a shady, well-ventilated place and extracted with ethanol (96%) at 50°C for 4 hrs. After filtration, the solvent was evaporated under reduced pressure, and the residue (grape pomace extract) was kept at −20°C until the time of the experiment. The exact polyphenolic composition of the tested extract has been determined by Veskoukis *et al.* [28].

#### Computer Assisted Sperm Analysis (CASA) for motility parameters

A sample from each incubation tube was subjected to motion analysis. At the initial time point, 10 μl from each sample were transferred onto a warmed slide and covered with a cover slip (22 × 22 mm) at 37°C. The CASA system consisted of a triocular optical phase contrast microscope (Nicon Eclipse E200; Nikon, Tokyo, Japan), equipped with a warming stage (Tokai, Japan) at 37°C and a Basler Scout CCD digital camera (Basler Vision Technologies, Ahrensburg, Germany). The camera was connected to a computer. For each treatment, two samples and 5 fields per sample taken at random were recorded and analyzed using Integrated Semen Analysis System software (ISAS MvCo, Valencia, Spain). Sampling was carried out using a 20× negative phase contrast objective. Image sequences were saved and analyzed afterwards. The standard parameter settings were as follows: 25 frames sec^−1^, 20–90 μm^2^ for head area, VCL >10 μm sec^−1^ to classify a spermatozoon as motile. For each sample, the following characteristics of sperm motility were determined: percentages of motile (“Rapid”, “Medium”, “Slow”), and progressively motile spermatozoa; percentage of total motile spermatozoa, three velocity parameters (VCL, velocity according to the actual path; VSL, velocity according to the straight path; VAP, velocity according to the smoothed path) and the amplitude of the lateral displacement of the sperm head (ALH) [34].

### One-step eosin-nigrosin staining technique for viability and acrosomal status

Additionally, a 5 μl sample from the control and four different grape extract concentrations were stained with 10 μl of eosin Y/nigrosin. Smears of spermatozoa in duplicates were prepared, according to this technique [35], immediately after thawing (time point 0 hr) and at 2 hrs and 4 hrs incubation at 37°C. Forty smears of sperm were tested and 200 spermatozoa were counted from each slide. Stained smears were evaluated according to their viability and acrosomal status and classified as alive and dead spermatozoa with intact or non-intact acrosome.

### Measurement of lipid peroxidation

Lipid oxidation was assessed by the TBARS (ThioBarbituric Acid Reactive Substances) assay on the basis of MDA formation [36]. MDA, the compound used as an index of lipid peroxidation, was determined by a selective third-order derivative spectrophotometric method (Shimadzu Model UV-160A), slightly modified to spermatozoa. In brief, 10^7^ washed spermatozoa which have been previously treated or not with different concentration of a polyphenol-rich grape pomace extract (1 μg ml^−1^, 2 μg ml^−1^, 5 μg ml^−1^) were mixed with 50 μl FeSO_4_ 7H_2_O (5 mM) and 2900 μl distilled water for 1 hr and incubated for 1 hr at 37°C. After 2 hrs and 4 of hrs of total incubation, the samples were mixed with 500 μl trichloroacetic acid 35% and 2000 μl butylated hydroxytoluene (BHT) in hexane and were centrifuged at 2000 g for 1 min. The top hexane layer was discarded and the bottom aqueous layer (2500 μl) was transferred to another tube containing 1500 μl thiobarbituric acid (TBA). After 30 min of incubation (70°C), the tubes were allowed to cool under tap water, and submitted to third-order derivative spectrophotometry. The concentration of MDA (ng per 10^7^ spermatozoa) was calculated on the basis of the height of the third-order derivative peak at 521.5 nm by referring to slope and intercept data of the computed least squares fit of a standard calibration curve prepared using 1,1,3,3-tetrahethoxypropane.

### Statistical analysis

The parameters: “Rapid”, “Progressive Motility”, “Total motility”, VCL, VSL, VAP and ALH were analyzed as repeated measurements using the LS Means Differences Tukey HSD-test (*p* <0.05). The percentage of sperm viability was analyzed by the Chi-Square Goodness of Fit Test for a Poisson distribution (*p* <0.05). Repeated measures ANOVA with the Bonferroni correction were used for statistical analysis of MDA formation, where the interaction between different concentration and time points was analyzed (*p* <0.05).
